# Concentration effects of grape seed extracts in anti-oral cancer cells involving differential apoptosis, oxidative stress, and DNA damage

**DOI:** 10.1186/s12906-015-0621-8

**Published:** 2015-03-29

**Authors:** Ching-Yu Yen, Ming-Feng Hou, Zhi-Wen Yang, Jen-Yang Tang, Kun-Tzu Li, Hurng-Wern Huang, Yu-Hsuan Huang, Sheng-Yang Lee, Tzu-Fun Fu, Che-Yu Hsieh, Bing-Hung Chen, Hsueh-Wei Chang

**Affiliations:** Department of Oral and Maxillofacial Surgery Chi-Mei Medical Center, Tainan, Taiwan; School of Dentistry, Taipei Medical University, Taipei, Taiwan; Cancer Center, Kaohsiung Medical University Hospital, Kaohsiung Medical University, Kaohsiung, Taiwan; Institute of Clinical Medicine, Kaohsiung Medical University, Kaohsiung, Taiwan; Kaohsiung Municipal Ta-Tung Hospital, Kaohsiung, Taiwan; Department of Biotechnology, Kaohsiung Medical University, Kaohsiung, Taiwan; Department of Radiation Oncology, Faculty of Medicine, College of Medicine, Kaohsiung Medical University, Kaohsiung, Taiwan; Department of Radiation Oncology, Kaohsiung Medical University Hospital, Kaohsiung, Taiwan; Department of Biomedical Science and Environmental Biology, Kaohsiung Medical University, Kaohsiung, Taiwan; Institute of Biomedical Science, National Sun Yat-Sen University, Kaohsiung, Taiwan; School of Dentistry and Wan-Fang Hospital, Taipei Medical University, Taipei, Taiwan; Department of Medical Laboratory Science and Biotechnology, School of Medicine, National Cheng Kung University, Tainan, Taiwan; Institute of Medical Science and Technology, National Sun Yat-sen University, Kaohsiung, Taiwan; Research Center of Environmental Medicine, Kaohsiung Medical University, Kaohsiung, 80708 Taiwan

**Keywords:** GSE, Apoptosis, Oxidative stress, DNA damage, Oral cancer

## Abstract

**Background:**

Grape seeds extract (GSE) is a famous health food supplement for its antioxidant property. Different concentrations of GSE may have different impacts on cellular oxidative/reduction homeostasis. Antiproliferative effect of GSE has been reported in many cancers but rarely in oral cancer.

**Methods:**

The aim of this study is to examine the antioral cancer effects of different concentrations of GSE in terms of cell viability, apoptosis, reactive oxygen species (ROS), mitochondrial function, and DNA damage.

**Results:**

High concentrations (50–400 μg/ml) of GSE dose-responsively inhibited proliferation of oral cancer Ca9-22 cells but low concentrations (1–10 μg/ml) of GSE showed a mild effect in a MTS assay. For apoptosis analyses, subG1 population and annexin V intensity in high concentrations of GSE-treated Ca9-22 cells was increased but less so at low concentrations. ROS generation and mitochondrial depolarization increased dose-responsively at high concentrations but showed minor changes at low concentrations of GSE in Ca9-22 cells. Additionally, high concentrations of GSE dose-responsively induced more γH2AX-based DNA damage than low concentrations.

**Conclusions:**

Differential concentrations of GSE may have a differentially antiproliferative function against oral cancer cells via differential apoptosis, oxidative stress and DNA damage.

## Background

Betel quid chewing is one of the main causes leading to oral cancer in Taiwan [1]. Arecoline, one of main effective components in betel quid, was reported to lead to DNA damage and apoptosis through the formation of reactive oxygen species (ROS) and contribute to oral carcinogenesis [2-5]. Therefore, the modulation of ROS level may be helpful for oral cancer prevention and therapy.

Grape seed extract (GSE) is a common dietary health supplement due to its natural ROS modulating ability [6]. Commercial preparations of GSE are marketed in the world as a dietary health supplements due to their natural free radical scavenging ability [6]. The cancer chemoprevention and anticancer potential of GSE has been well reviewed previously [7] including skin, colorectal, prostate, breast, lung, and gastric cancers. However, the GSE effects with respect to oral cancer cells are less studied as yet.

ROS modulation effect has been well reviewed [8,9]. For example, cellular ROS may regulate apoptosis through the mitochondrial pathway [10-13]. Pro-oxidants induce ROS specifically targeting cancer cells, thereby activating signal transduction pathways that are responsible for cell cycle arrest and/or apoptosis [14]. Similarly, GSE was reported to generate a strong superoxide radical-associated oxidative stress and result in the apoptosis of non-small-cell lung cancer cells [15] as well as in the induction of DNA damage [16].

Different concentrations of GSE were reported to generate diverse biological effects in several cancer studies [17-20]. For example, high concentrations (25–100 μg/ml) of GSE showed cytotoxicity or antiproliferation of human bladder [17], colorectal [21], and breast [18] cancer cell lines. In contrast, a low concentration (2.5 μg/ml) of GSE was reported to inhibit the micronuclei frequency and ROS generation in a lymphocyte culture, demonstrating that its antioxidant property has a protective effect during oxidative stress [19]. However, more detailed mechanisms between cancer chemoprevention and anticancer effects of GSE in terms of concentration effects remain unclear.

Since GSE is a natural ROS scavenger, we hypothesize that GSE modulates ROS to further regulate proliferation, apoptosis, mitochondrial function, and DNA damage. Since concentration responses of GSE for these regulations may be relevant, in this study we aim to define the critical concentrations that may or may not be able to induce apoptosis in oral cancer cells.

## Methods

### GSE source

The IH636 premium grade proanthocyanidin grape seed (*Vitis vinifera*) extract (GSE, commercially known as ActiVin®) was purchased from InterHealth Nutraceuticals Inc. (Benicia, CA, USA), which included 75–80% oligomeric proanthocyanidins and 3–5% monomeric proanthocyanidins as described previously [22].

### Cell cultures

Cell lines of human oral gingival cancer Ca9-22 [23] and gingival fibroblast HGF-1 [24] were routinely maintained in DMEM/F12 medium (Gibco, Grand Island, NY) containing 10% fetal bovine serum, 0.03% glutamine, 1 mM sodium pyruvate, and penicillin/streptomycin mixtures. Cells were kept at 37°C in a humidified incubator containing 5% CO_2_.

### Determination of cell viability

Viability analysis was performed using Cell Titer 96™ Aqueous One solution cell proliferation (3-(4,5-dimethylthiazol-2-yl)-5-(3-carboxymethoxyphenyl)-2-(4- sulfophenyl)-2H-tetrazolium) MTS) assay kit (Promega Madison, WI, USA) as described previously [25] with minor modification. In brief, cells were treated with various concentrations of GSE in fresh media in triplicates. The non-toxic concentration of DMSO (less than 1% v/v) was used to prepare test solutions in all assays. The plates were then incubated for 24 h under standard growth conditions. Subsequently, MTS reagent was loaded to each well (5 mg/ml in PBS) and cells were again incubated for another 2 h. Then, absorbance of each well was recorded directly at 490 nm by ELISA multi-Plate Reader (MTX Lab Systems, Inc., Vienna, VA, USA).

### Determination of sub-G1 population

Measurement of DNA content for cell cycle analysis were carried out by flow cytometry, based on a previously described protocol [26]. In brief, Ca9-22 cells were treated with either DMSO only or different GSE concentrations for 24 h. After incubation, cells were harvested for washing and fixing in 70% ethanol overnight. After harvest, cells were resuspended in 1 ml PBS containing 10 μg/ml PI (Sigma, St Louis, MO, USA) in the dark. Subsequently, cells were analyzed using a flow cytometer (FACScan; Becton-Dickinson, Mansfield, MA) at excitation and emission settings of 480 and 525 nm, respectively, and Win-MDI software (http://facs.scripps.edu/software.html).

### Determination of apoptosis by annexin V/PI

The induction of apoptosis by GSE-treated Ca9-22 cells was analyzed by annexin V staining as previously described [27]. Briefly, cells were treated with either vehicle or various GSE concentrations for 24 h. Subsequently, the cells were trypsinized, washed twice with PBS and stained with fluorescein isothiocyanate (FITC)-labelled annexin V. Then, the samples were measured with a flow cytometer (FACSCalibur; Becton-Dickinson) for the quantification of apoptotic cells at excitation and emission settings of 480 and 525 nm, respectively, and Win-MDI software.

### Determination of apoptosis by pan-caspase activity

The induction of apoptosis by GSE-treated Ca9-22 cells was analyzed by activation of caspases (caspase-1, 3, 4, 5, 6, 7, 8, 9) by the generic caspase activity assay kit (Abcam, Cambridge, UK) as previously described [27]. Briefly, cells were treated with either vehicle or various GSE concentrations for 24 h. After harvest, the cells were suspended and stained with 1 X fluorescent TF2-Val-Ala-Asp (VAD)-FMK at the cell incubator for 1 h. Then, the samples were measured with a flow cytometer (BD Accuri C6; Becton-Dickinson, Mansfield, MA, USA) and a BD Accuri C6 Software (version 1.0.264) for the quantification of pan-caspase positive populations at excitation and emission settings of 480 and 525 nm, respectively.

### Determination of intracellular ROS

Intracellular redox state were determined by the ROS-sensitive dye 2′,7′-dichlorodihydrofluorescein diacetate (DCFH-DA) (Sigma Chemical Co., St. Louis, MO, USA) as previously described [25,28]. Ca9-22 cells were treated with various concentrations of GSE for 24 h. Subsequently, cells were harvested, thoroughly washed, resuspended in 10 μM DCFH-DA in PBS and then incubated at 37°C for 30 min in darkness. After incubation, cells were washed, resuspended in PBS, and analyzed with a FACSCalibur flow cytometer at excitation and emission settings of 480 and 525 nm, respectively, and Win-MDI software.

### Determination of mitochondrial membrane potential

Mitochondrial membrane potential (MitoMP) was determined by flow cytometry using MitoProbe™ DiOC2(3) assay kit (Invitrogen, San Diego, CA, USA) as described previously [25]. In brief, cells were incubated with various GSE concentrations at 37°C for 24 h. Subsequently, cells were incubated in culture medium (containing 50 μM of DiOC_2_(3)) at 37°C for 20 min in an incubator. After washing and resuspension in PBS, cells were subjected to flow cytometric analysis. The fluorescence intensity was measured using 488 and 525 nm filter settings for the excitation and emission wavelengths, respectively. The data were analyzed with Win-MDI software.

### Determination of DNA double strand breaks (DSBs) by γH2AX/PI double staining

DSBs were measured by flow cytometry as described previously [25]. Ca9-22 cells were incubated with various GSE concentrations for 24 h, followed by fixation with 70% ethanol overnight. After washing twice with BSA-T-PBS (1% bovine serum albumin and 0.2% Triton X-100 in PBS), cells were treated with 100 μl of BSA-T-PBS solution containing 0.2 μg monoclonal antibody against p-Histone H2A.X (Ser 139) (Santa Cruz Biotechnology, Santa Cruz, CA, USA) for overnight at 4°C. After washing, cells were resuspended in Alexa Fluor 488-tagged secondary antibody (Jackson ImmunoResearch Laboratories, Inc., West Grove, PA, USA) at a 1:100 dilution for 1 h at 4°C. After washing, cells were resuspended in 1 ml PBS containing 5 μg/ml PI and analyzed by a FACSCalibur flow cytometer and Win-MDI software.

### Statistical analysis

Statistical analysis was performed with JMP 9 software. One-way ANOVA with Tukey’s HSD Post Hoc Test was used to analyze significant differences between treatments. Unless otherwise indicated, all experiments were repeated in triplication.

## Results

### Cell viability

**Figure 1:**
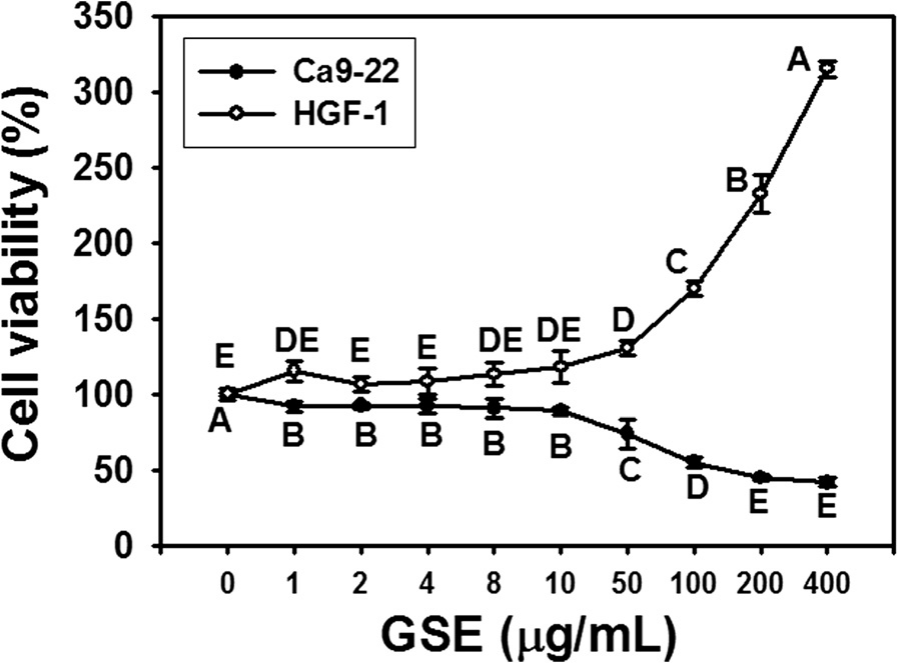
**Cytotoxicity of GSE treatments on oral cancer Ca9-22 cells and normal oral HGF-1 cells.** Cells were treated with either vehicle control (DMSO) or with 2, 4, 8, 10, 50, 100, 200 and 400 μg/ml of GSE for 24 h. Cell viability was detected by the MTS assay. The percent cell viability in the experimental groups was adjusted to the DMSO-treated group representing 100% viability. Data, mean ± SD (n = 10 and 5 for Ca9-22 and HGF-1 cells, respectively). Treatments with the same capital letter are nonsignificant.

To access the potential harmful effects of GSE on Ca9-22 cells, cell viability was determined by MTS reagent. At low concentrations of GSE treatment (1–10 μg/ml), the cell viabilities maintained around 91% (Figure 1). In contrast, a significantly concentration-dependent decrease in cell viability (*P* < 0.005–0.0001) was observed at high GSE concentrations (50, 100, 200 and 400 μg/ml). The IC_50_ of GSE for Ca9-22 cells was 150 μg/ml at 24 h incubation. However, both the low and high concentrations of GSE were not harmful to normal oral HGF-1 cells.

### Cell cycle distribution by GSE treatments

**Figure 2:**
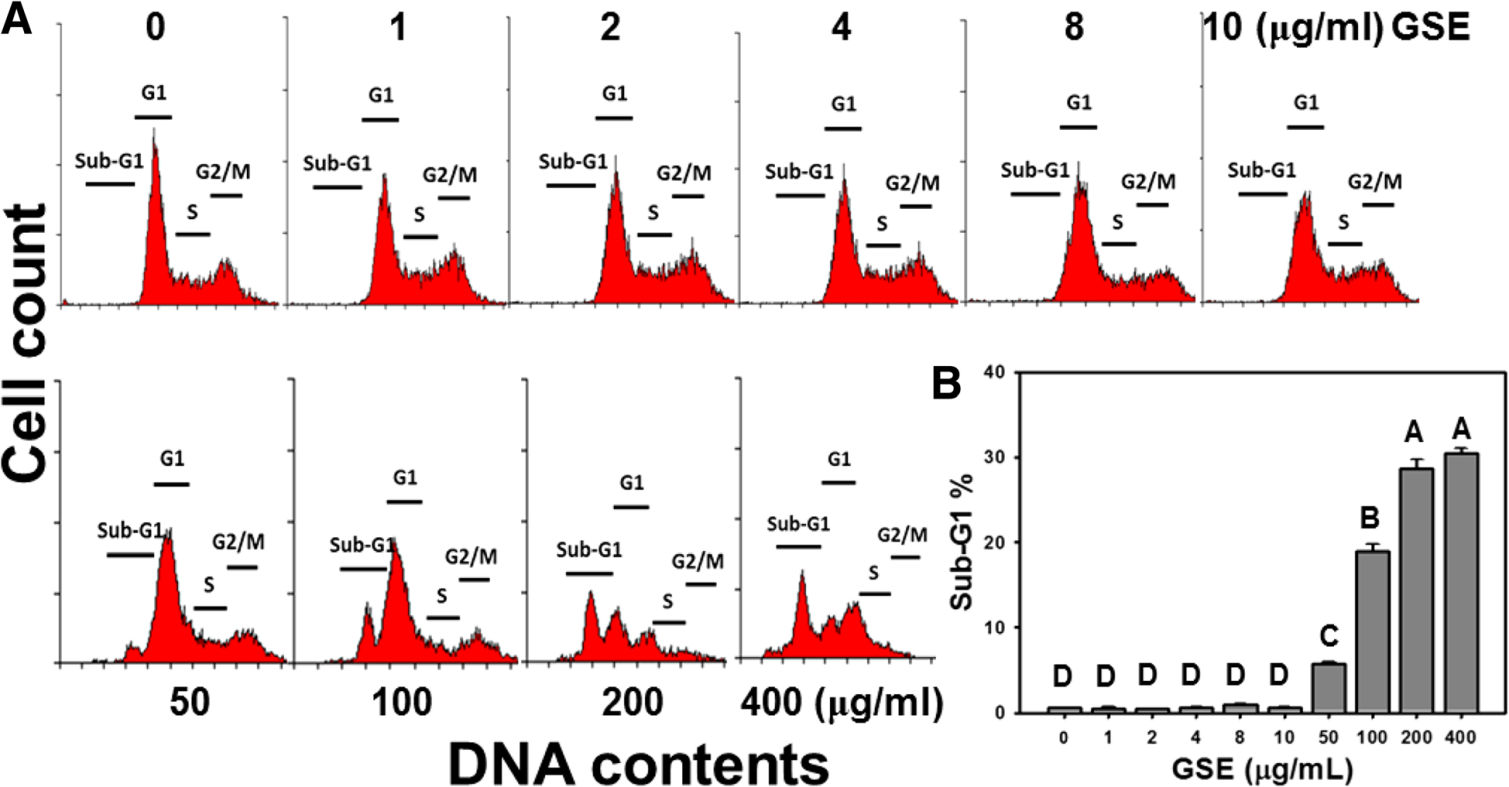
**Analysis on distribution of cell cycle in GSE-treated Ca9-22 cells.** Ca9-22 cells were treated with indicated GSE concentrations (0–400 μg/ml) for 24 h before being harvested, fixed and stained with PI for cell cycle analysis. **(A)** Representative histograms for cell cycle phases in GSE-treated Ca9-22 cells. **(B)** Quantitative analysis on distribution of cell cycle phases. Data, mean ± SD (n = 3). Treatments with the same capital letter are nonsignificant.

To investigate if GSE treatments cause change in cell cycle distribution in Ca9-22 cells, a standard PI-staining protocol was applied to GSE-treated Ca9-22 cells. In Figure 2A, the cell cycle distributions were stable at low concentrations (1–10 μg/ml) of GSE but the subG1 populations were gradually accumulated at high concentrations of GSE. In Figure 2B, the change in the sub-G1 populations (%) of Ca9-22 cells was not significant at low concentrations of GSE. However, the changes in the sub-G1 populations (%) significantly increased to 5.61, 16.73, 25.69 and 26.80 in a concentration-dependent manner (*P* < 0.0001) when GSE concentrations were increased at 50, 100, 200 and 400 μg/ml, respectively. Additionally, the percentage changes in other cell cycle phases (i.e. the G1, S and G2/M phases) did not exhibit significant changes in all treatment groups compared to untreated samples.

### Apoptotic cell death: annexin V/PI

**Figure 3:**
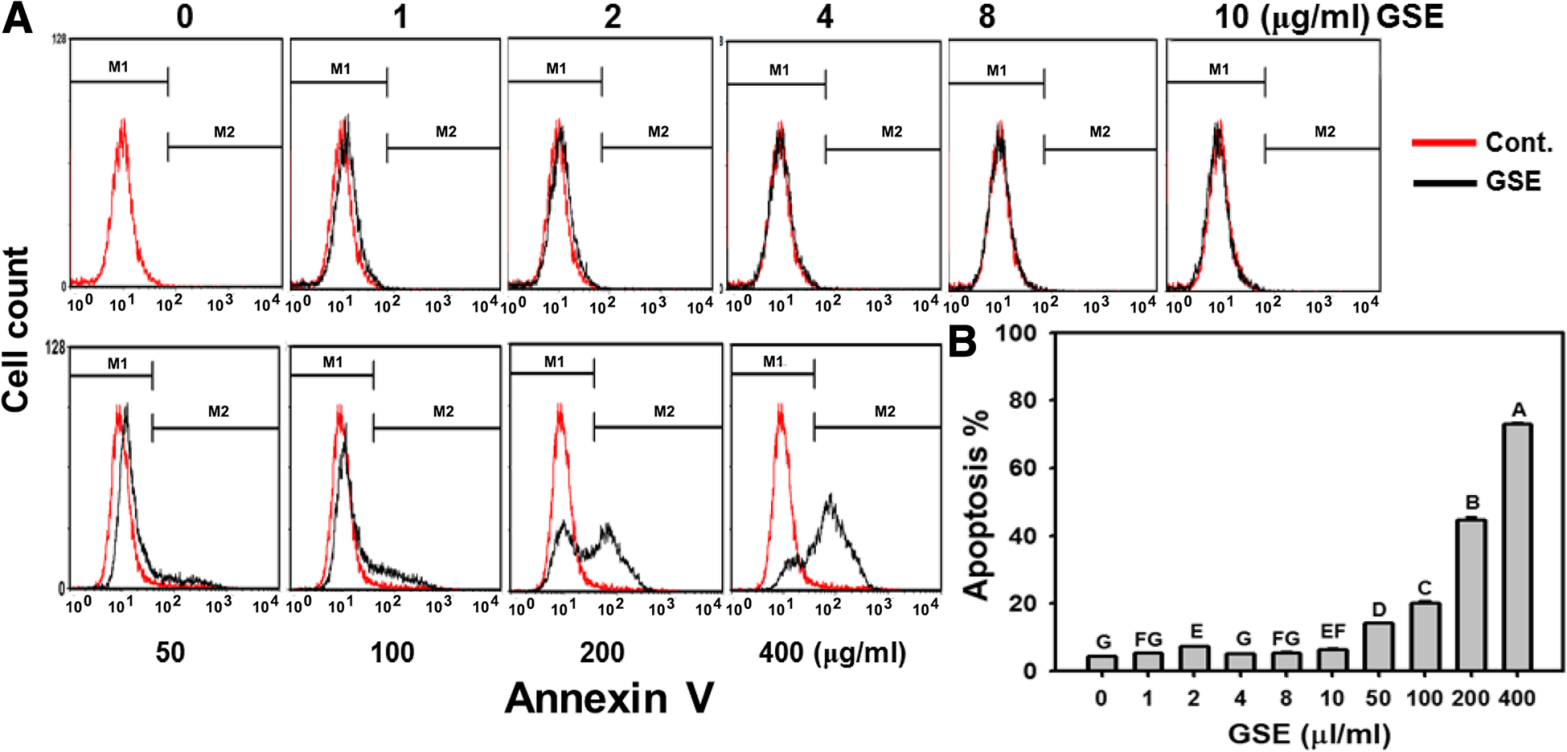
**GSE induced annexin V/PI-based apoptosis in Ca9-22 cells.** Cells were treated with indicated GSE concentrations (0–400 μg/ml) for 24 h. **(A)** Histograms of representative annexin V-FITC profile in GSE-treated Ca9-22 cells. **(B)** Quantitative analysis for the apoptotic cells (%). Apoptosis was counted at the intensity of right gated region in **(A)**. Data, mean ± SD (n = 3). Treatments with the same capital letter are nonsignificant.

To determine the degree of apoptosis of GSE-induced cell death in Ca9-22 cells, the annexin V-FITC staining was determined by flow cytometry. In Figure 3A, the apoptosis signals were similar at low concentrations (1–10 μg/ml) of GSE but they gradually increased at high concentrations of GSE. In Figure 3B, the annexin V intensity of GSE-treated Ca9-22 cells was weak at low concentrations of GSE. However, the percentage changes in the annexin V intensity significantly increased to 14.19%, 20.03%, 44.53, and 72.86% in a concentration-dependent manner at high concentrations (50, 100, 200 and 400 μg/ml) of GSE (*P* < 0.05–0.0001).

### Apoptotic cell death: caspase activity

**Figure 4:**
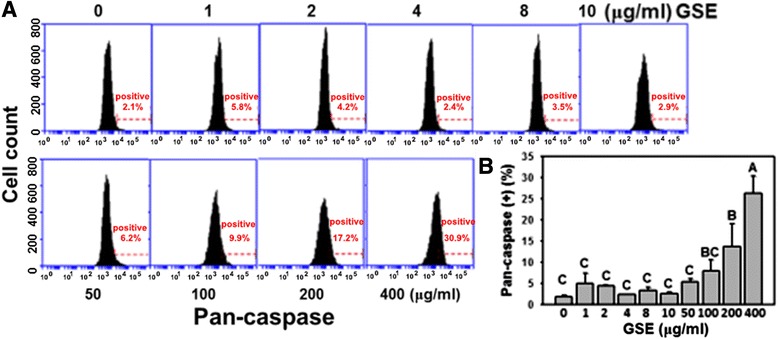
**GSE induced pan-caspase-based apoptosis in Ca9-22 cells.** Cells were treated with indicated GSE concentrations (0–400 μg/ml) for 24 h. **(A)** Histograms of representative pan-caspase activity profile in GSE-treated Ca9-22 cells. Caspase positive % was indicated in the right gated region of each panel. **(B)** Quantitative analysis for the pan-caspase positive (%). Data, mean ± SD (n = 3). Treatments with the same capital letter are nonsignificant.

To further determine the degree of apoptosis of GSE-induced cell death in Ca9-22 cells, the multiple caspase (pan-caspase) activity staining was determined by flow cytometry. In Figure 4A, the apoptosis signals based on pan-caspase intensities were similar at low concentrations (1–10 μg/ml) of GSE but they gradually increased at high concentrations of GSE. In Figure 4B, the pan-caspase positive intensity of GSE-treated Ca9-22 cells was weak at low concentrations of GSE. However, the percentage changes in the pan-caspase positive intensity concentration-responsively increased at high concentrations (50, 100, 200 and 400 μg/ml) of GSE, respectively (*P* < 0.01–0.001).

### Intracellular ROS

**Figure 5:**
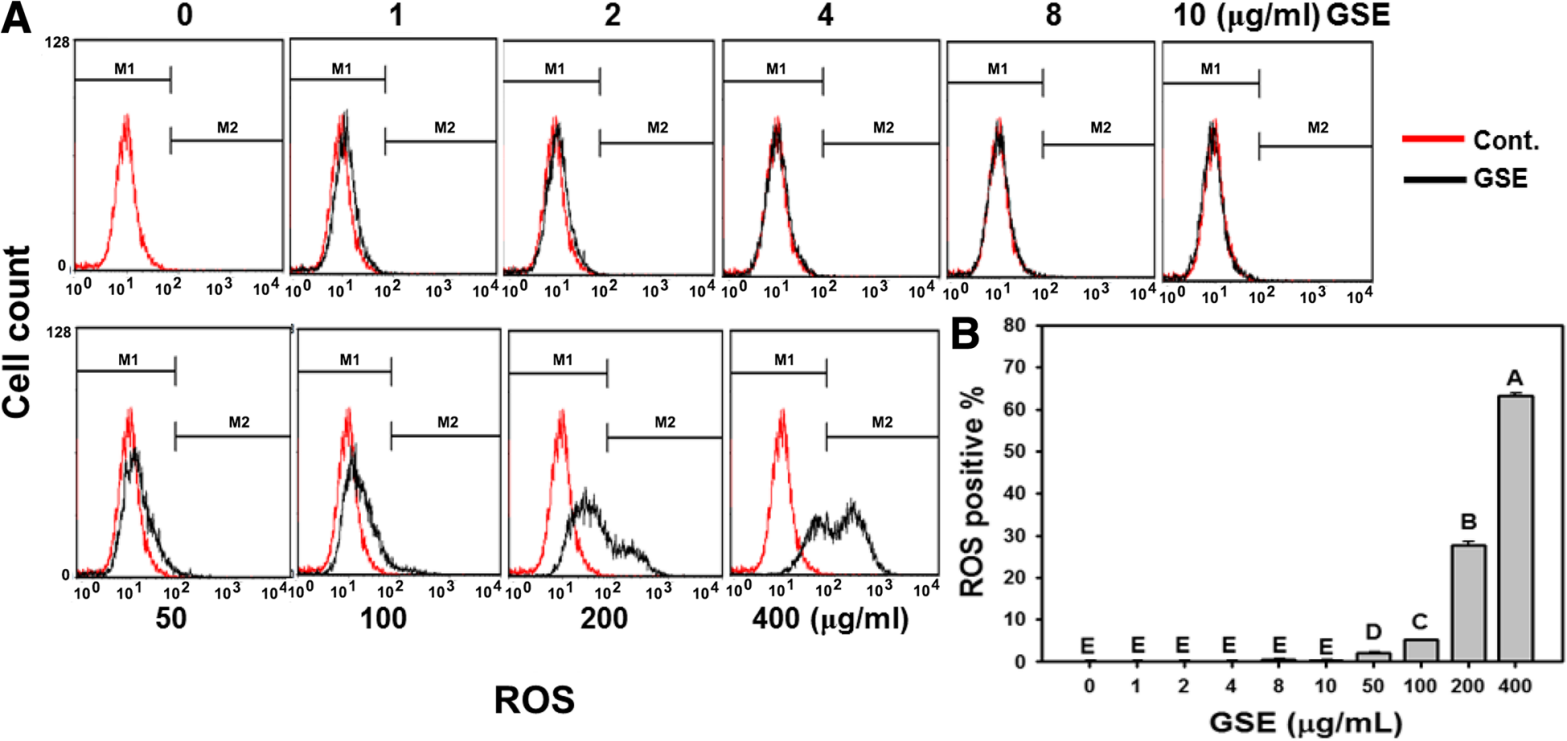
**Concentration-dependent ROS generation in GSE-treated oral cancer Ca9-22 cells.** Ca9-22 cells were incubated with indicated concentrations of GSE (0–400 μg/ml) for 24 h. **(A)** Representative histograms of flow cytometric analysis on ROS levels from GSE-treated cells. **(B)** Quantitative analysis of ROS intensity by means of DCF positivity percentage. ROS is counted at the intensity of right gated region in **(A)**. Data, mean ± SD (n = 3). Treatments with the same capital letter are nonsignificant.

ROS plays a pivotal role in regulating cellular apoptosis. To determine whether Ca9-22 cells exposed to GSE bore higher levels of ROS, we monitored the intracellular ROS levels using the fluorescent dye DCFH-DA as a specific ROS scavenger. In Figure 5A, the ROS signals were similar at low concentrations (1–10 μg/ml) of GSE but they were gradually increased at high concentrations of GSE. In Figure 5B, no significant elevation of ROS levels became apparent in Ca9-22 cells treated with GSE concentrations lower than 10 μg/ml. However, after exposing Ca9-22 cells to GSE at 50, 100, 200 and 400 μg/ml for 24 h, the ROS levels increased significantly in a concentration-dependent manner to 2.10%, 5.10%, 27.77%, and 63.17%, respectively (*P* < 0.005–0.0001).

### MitoMP

**Figure 6:**
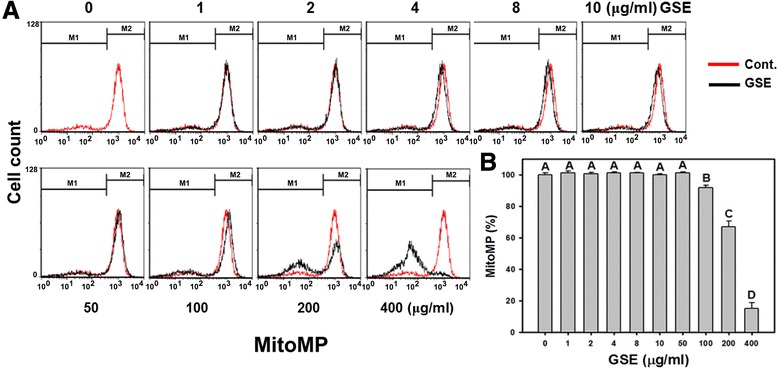
**Reduction of MitoMP in GSE-treated Ca9-22 cells.** Ca9-22 cells were treated with indicated GSE concentrations (0–400 μg/ml) for 24 h before the addition of fluorescent dye Rh123 (0.5 μg/ml) for 30 min. **(A)** Representative histograms on MitoMP levels for GSE-treated Ca9-22 cells. **(B)** Quantitative analysis on reduction of MitoMP in vehicle controls and GSE-treated cells. MitoMP is counted at the intensity of the left gated region in **(A)**. Data, mean ± SD (n = 3). Treatments with the same capital letter are nonsignificant.

To examine the involvement of GSE-induced mitochondrial dysfunction in Ca9-22 cells, the flow cytometry-based Rh123 staining was performed. In Figure 6A, the mitoMP signals were similar in low concentrations (1–10 μg/ml) of GSE but they were gradually decreased in high concentrations of GSE after 100 μg/ml of GSE. In Figure 6B, there was not a significant elevation of mitoMP levels in Ca9-22 cells incubated with GSE concentrations lower than 10 μg/ml. In contrast, after exposing Ca9-22 cells to GSE at 50, 100, 200 and 400 μg/ml for 24 h, the mitoMP levels were significantly decreased in a concentration-dependent manner to 101.14%, 91.69%, 66.97%, and 15.01%, respectively (*P* < 0.005–0.0001).

### DNA damages caused by GSE treatment

**Figure 7:**
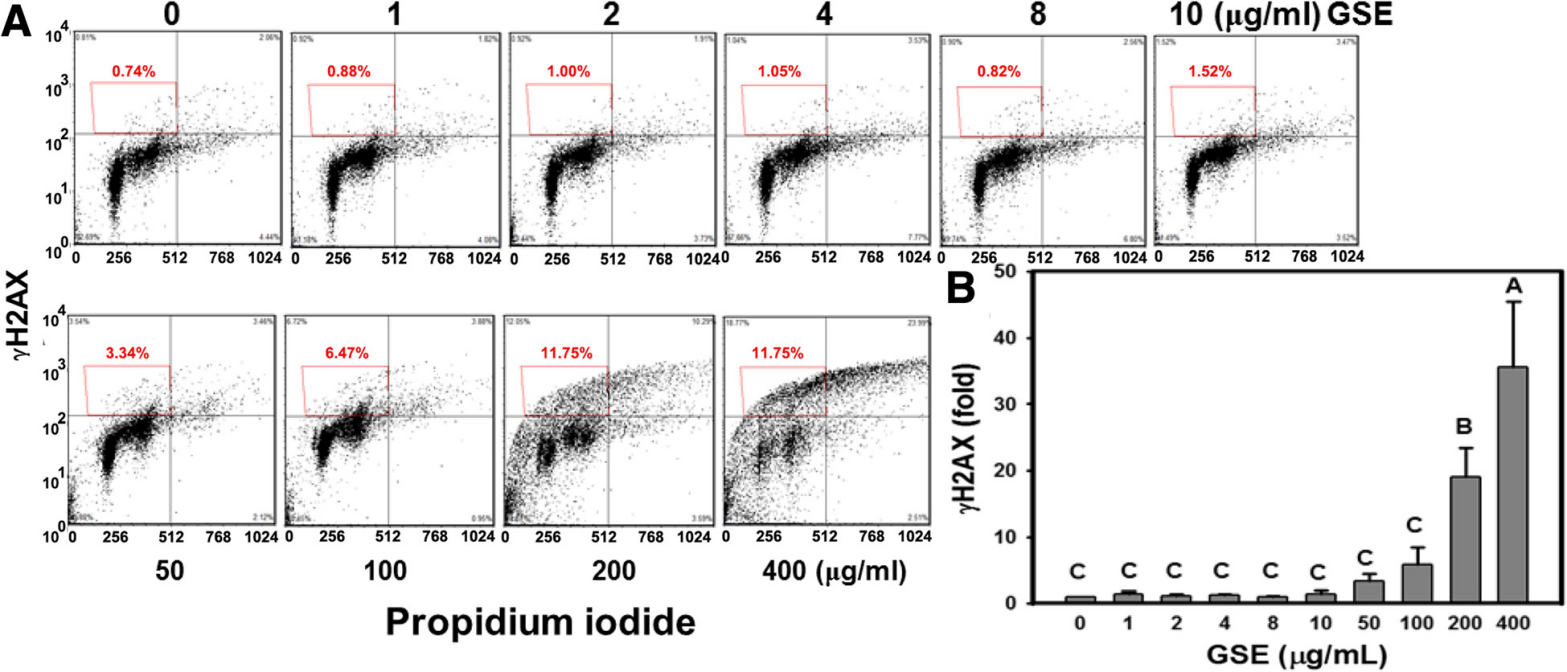
**GSE treatments caused γH2AX-based DNA damage in Ca9-22 cells.** Cells were treated with different GSE concentrations for 24 h before subjected to anti-phospho-γH2AX/PI double staining for measuring DNA double strand breaks by flow cytometry.** (A)** A representative γH2AX/PI profile for GSE-treated cells.** (B)** Quantitative analysis of γH2AX-based DNA damage in GSE-treated cells. The mean fluorescence intensity of phospho-γH2AX for gated regions in **(A)** was calculated. Data, mean ± SD (n = 3). Treatments with the same capital letter are non-significant.

To detect whether GSE treatments cause DNA double strand break (DSB) in Ca9-22 cells, samples were analyzed using flow cytometry to quantify levels of the phosphorylated γH2AX protein. In Figure 7A, the γH2AX signals were similar in low concentrations (1–10 μg/ml) of GSE but they were gradually increased in high concentrations of GSE. In Figure 7B, there was not a significant elevation of γH2AX levels in GSE-treated Ca9-22 cells under low concentrations (lower than 10 μg/ml). In contrast, after exposing Ca9-22 cells to GSE at 50, 100, 200 and 400 μg/ml for 24 h, the γH2AX levels were significantly increased in a concentration-dependent manner to 3.38%, 5.88%, 19.02%, and 35.53%, respectively (*P* < 0.001).

## Discussion

Accumulating evidence of the antiproliferative effect of GSE had been reported in several oral cancer cell lines. For example, high concentrations (50–600 μg/ml) of GSE of *Vitis vinifera* were found to inhibit cell proliferation and induce apoptosis of the KB cells but less harmful to non-cancerous human umbilical vein endothelial cells (HUVEC) by trypan blue assay at 24 h GSE treatment [29]. Similarly, we found that the low and high concentrations of GSE to normal oral HGF-1 cells based on MTS analysis. The KB cells was used to be regarded as the oral cancer cell line, however, it was recently confirmed to be the contaminant cervical cancer HeLa cells [30]. Moreover, the low concentrations of GSE were not investigated in this study. Recently, the differential concentration effect of GSE to differentially inhibit proliferation of oral cancer cells has been demonstrated. For example, low concentrations of GSE (10–20 μg/ml) did not displayed the antiproliferation of oral cancer CAL 27 cells but high concentrations of GSE (30–80 μg/ml) were able to inhibit its proliferation [31]. Similarly, we found that low (1–10 μg/ml) and high (50–400 μg/ml) concentrations of GSE displayed the differential cytotoxic effects to cell viability of oral cancer Ca9-22 cells. Similar results also reported in other cancer cells. In the example of skin cancer HaCaT cells, high concentrations of GSE (IC_50_ = 76.44 μg GAE/ml) displayed the growth inhibitory effect, but low concentrations of GAE (10–20 μg GAE/ml) protected against UVB irradiation (50–100 mJ/cm^2^)-induced skin cancer [20]. These findings suggested that different cancer cell lines may require different but high concentrations of GSE for antiproliferation purpose.

ROS induction by GSE was reported in non-small-cell lung cancer H1299 and A549 cells but it only tested at high concentrations (20–100 μg/ml) without detecting the mitochondrial function [15]. ROS generation of high GSE (40 μg/ml) also reported to induce apoptosis in head and neck cancer Detroit 562 and FaDu cells [32]. In oral cancer CAL 27 cells, GSE also reported to induce mRNA overexpression of apoptosis-associated signaling such as caspase-2 and caspase-8 [31]. In head and neck cancer cells, GSE also reported to induce DNA damage [32]. Our results further validated that GSE at high concentrations (50–400 μg/ml) have high oxidative stress and apoptosis in terms of ROS generation, mitochondrial depolarization, annexin V/PI staining, and caspase activation but not for low concentrations (<10 μg/ml) of GSE in oral cancer Ca9-22 cells.

Moreover, this differential concentration effect of GSE was also found in cancer cell migration. For example, GSE was reported to inhibit migration and invasion of breast cancer MDA-MB231 cell [18]. High concentrations (50–100 μg/ml) of GSE inhibited cell proliferation and induced apoptosis. Conversely, low GSE (25 μg/ml) concentrations decreased cell migration and invasion. Therefore, the differential concentration effect of GSE in oral cancer cell migration is warranted for further investigation.

## Conclusion

We demonstrated that GSE shows differential concentration effects in the antiproliferation of oral cancer cells through differential expressions of apoptosis, oxidative stress, and DNA damage. We showed that the antiproliferative effect of high GSE concentrations is associated with an overproduction of ROS causing DNA damage and apoptosis of cancer cells.
